# Genetic polymorphisms related to the vitamin D pathway in patients with cirrhosis with or without hepatocellular carcinoma (HCC)

**DOI:** 10.3332/ecancer.2022.1383

**Published:** 2022-05-04

**Authors:** Beatriz de Jesus Brait, Simone Perpétua da Silva Lima, Franciana Luísa Aguiar, Rafael Fernandes-Ferreira, Camila Ive Ferreira Oliveira-Brancati, Joyce Aparecida Martins Lopes Ferraz, Graciele Domitila Tenani, Marcela Augusta de Souza Pinhel, Leticia Carolina Paraboli Assoni, Augusto Haniu Nakahara, Natalia dos Santos Jábali, Octavio Pennella Fenelon Costa, Maria Eduarda Lopes Baitello, Sidney Pinheiro Júnior, Renato Ferreira Silva, Rita de Cássia Martins Alves Silva, Doroteia Rossi da Silva Souza

**Affiliations:** 1 São José do Rio Preto Medical School (FAMERP), São José do Rio Preto, SP, 15090-000, Brazil; 2 Campinas State University (UNICAMP), Campinas, SP, 13083-970, Brazil; 3 Paulista University (UNIP) São Paulo, SP, 01311-000, Brazil; a https://orcid.org/0000-0002-8901-3996

**Keywords:** liver, single nucleotide polymorphism, neoplasia, vitamin D

## Abstract

**Objective:**

To evaluate the association of genetic polymorphisms of vitamin D transporter protein (*DBPrs4588* and* DBP-rs7041*) and cytochrome P450-24A1 (*CYP24A1-rs6013897*) in patients with cirrhosis with or without hepatocellular carcinoma (HCC), including demographic/clinical/biochemical profiles.

**Methods:**

A total of 383 individuals were studied, considering the total group (TotalG) of patients with cirrhosis (TotalG: *N *= 158) with or without HCC, distributed into Group 1 (G1): cirrhosis and HCC; Group 2 (G2): isolated cirrhosis; and 225 individuals without hepatopathies (G3). Polymorphisms were analysed by real-time polymerase chain reaction. An alpha error of 5% was admitted.

**Results:**

*CYP24A1-rs6013897* predominated the genotype with at least one polymorphic allele (_/T) in G1 (98.3%) versus G2 (88.8%; *p *= 0.0309). There was a moderate positive correlation between vitamin D and parathyroid hormone in patients (TotalG: *R*^2 ^= 0.3273). Smoking, alcoholism and diabetes mellitus (DM) stood out as independent factors for cirrhosis, as well as for cirrhosis with HCC, except for smoking, adding, in this case, advanced age, male gender, polymorphic allele of *CYP24A1-rs6013897*, viral hepatitis and high levels of serum gamma-glutamyl transferase (GGT), alpha-fetoprotein (AFP) and creatinine. An increase in survival was observed in the presence of the polymorphic allele of *DBP-rs7041* (*p *= 0.0282).

**Conclusion:**

*CYP24A1-rs6013897* is associated with cirrhosis and HCC as a predictor, while *DBP-rs4588 *is associated with reduced vitamin D, and *DBP-rs7041 *provides increased survival, suggesting a protective characteristic. Advanced age, alcoholism, DM, viral hepatitis and high levels of GGT, AFP and creatinine are also confirmed as predictors of HCC and cirrhosis, while smoking, alcoholism and DM for isolated cirrhosis only.

## Introduction

Cirrhosis, the tenth leading cause of death in the world [1], is an irreversible chronic liver disease characterised by fibrosis, several structural liver lesions [2] and the formation of regenerative nodules [3], whose prevalence is underestimated in asymptomatic patients [4].

Cirrhosis is directly associated with the development of hepatocellular carcinoma (HCC) [2], present in 90% of the cases of this tumour type [5]. HCC represents the main primary liver neoplasia [6], being the second most common cause of cancer-related deaths [6] in the world, with 905,677 cases per year [7].

Admittedly, biomolecular events that trigger hepatic carcinogenesis may result from alterations that interfere with the synthesis of deoxyribonucleic acid (DNA) [8]. In addition, the role of vitamin D stands out, being recognised for maintaining calcium homeostasis, immunomodulation, participation as a modulator of cell proliferation and for its inhibitory power in cancer [9].

The vitamin D transporter protein gene (*DBP*) participates and functions in metabolic and biological pathways, such as the regulation of bone development, including actin capture and modulation of immune responses [10]. In this case, the genetic variants *DBP-rs4588* and *DBP*-*rs7041* are associated with reduced serum levels of 25-hydroxy-vitamin D [25(OH)D] [11], which may have clinical consequences [11], which need clarification for cirrhosis and HCC.

The gene of the cytochrome P450 from the family 24, subfamily A, member 1 (*CYP24A1*) encodes 25-hydroxyvitamin D_3_-24-hydroxylase. This enzyme catalyses the conversion of 25(OH)D_3_ and 1.25(OH)_2_D_3_ into hydroxylated products, which constitute the degradation of the vitamin D [12] molecule. Additionally, the increased expression of *CYP24A1*, a regulator of vitamin D, and the activity of the respective 24-hydroxylase enzyme are associated with the degradation of 25(OH)D_3_ into 24.25(OH)_2_D_3_ [13]. *CYP24A1* induces low serum levels of 25(OH)D_3_, which has been shown with liver disease severity in studies with non-alcoholic fatty liver disease (NAFLD) [14] and patients with hepatitis C (HCV) [15]. Furthermore, some studies suggest that *CYP24A1* single nucleotide polymorphisms (SNPs) contributes to a chronic HCV infection, progressing to more defined conditions [16], such as the risk for liver cancer [17].

It is noteworthy that vitamin D, in its active form 1.25(OH)_2_D, regulates several genes, including the key effector of its catabolism, the *CYP24A1* [18] related to prostate cancer [19], whose expression is increased in patients with advanced stage disease [20]. Furthermore, the genetic variants of *CYP24A1 (rs6013897)* are associated with inflammatory reactions [21], which should be clarified in liver diseases.

Thus, this study aimed at evaluating the association of genetic polymorphisms of *DBP (rs4588* and *rs7041*) and *CYP24A1 (rs6013897)* in cirrhosis with or without HCC, considering demographic–clinical–biochemical profile, as well as lifestyle and survival habits.

## Methods

A total of 383 individuals were studied and distributed into groups. The total group (TotalG), consisting of 158 patients, was subdivided into Group 1 (G1) = 60 (46–81 years), with a diagnosis of cirrhosis with HCC; and Group 2 (G2) = 98 (16–71 years), with isolated cirrhosis, both monitored at the Liver Transplant Service and Cancer Institute, Base Hospital/São José do Rio Preto Medical School-HB/FAMERP. A group of 225 individuals without clinical and laboratory evidence (serum levels of aspartate aminotransferase (AST), alanine aminotransferase (ALT) and gamma-glutamyl transferase (GGT)) of liver diseases – control group – (G3), aged 20–84 years, was selected at the Blood Centre – HB/FAMERP, in addition to the volunteers. This study was approved by the Research Ethics Committee of São José do Rio Preto Medical School (CEP/FAMERP; Process number 435/2011 and 6910/2011).

Exclusion criteria included diseases correlating with reduced serum levels of vitamin D, such as type 1 diabetes, Crohn’s disease, tuberculosis, Hansen’s disease, multiple sclerosis, autoimmune hepatitis, psoriasis, Graves’ disease, and the use of vitamin supplements with vitamin D.

The diagnosis of cirrhosis was made by the combination of clinical criteria present in the disease (reduced liver volume, irregular surface and increased consistency, splenomegaly and peripheral signs of chronic liver disease and portal hypertension), laboratory tests (plateletopenia, hypoalbuminemia and coagulation disorders) and imaging (reduced liver volume, irregular surface and signs of portal hypertension), and by biopsy, when necessary.

The diagnosis of HCC was carried out according to the practice guidelines of the American Association for the Study of Liver Disease [22]. The degree of staging for cirrhosis and HCC was classified using the Child-Pugh and model for end-stage liver disease methods, and both were used to classify the severity of liver disease [23].

Data on comorbidities (systemic arterial hypertension (SAH) and diabetes mellitus (DM)), lifestyle (smoking and alcohol consumption), demographic profile (gender and age) and biochemical profile (AST, ALT, GGT, alpha-fetoprotein (AFP), bilirubin and albumin) were obtained through a questionnaire, electronic or physical medical records and pre-established protocols for routine follow-up of patients in those services.

Serum dosages of vitamin D and parathyroid hormone (PTH) were measured in the subgroups: TGd = 30 patients from the TotalG, 4 with cirrhosis and HCC (G1d) and 26 with isolated cirrhosis (G2d); and G3d = 20 individuals in the control group without hepatopathies. Altered values for vitamin D, serum levels <30 ng/mL [24] and for PTH <15 and >65 pg/mL [25] were considered.

After genomic DNA extraction from the peripheral blood [26], SNPs of the* DBP-rs4588* (C___8278879_10), *DBP-rs7041* (C___3133594_30) and *CYP24A1-rs6013897* (C__29958084_10) genes were genotyped by *TaqMan***^®^** (Thermo Fisher) by polymerase chain reaction (PCR), according to the manufacturer’s manual. The reactions for the amplification of the polymorphic segments were carried out in the following thermocycles: 95°C for 10 minutes, followed by 47 cycles at 92°C for 15 seconds each and 60°C for 1 minute, in the StepOnePlus Real-Time PCR System (Applied Biosystems). Allele discrimination was conducted using the StepOne software v2.3 (Applied Biosystems) programme.

Fisher’s exact test or chi-square test was applied to analyse qualitative variables, including genotypic and allelic frequencies, sex, distribution of altered values for biochemical profile, comorbidities and lifestyle habits. *T*-test or Mann–Whitney test was used to analyse quantitative variables (age and biochemical profile) in the GraphPad Prism programme (version 5.0). Actuarial survival curve (Kaplan–Meier) was analysed considering at least one polymorphic allele, as well as logistic regression analysis (StatsDirect Statistical Analysis). For SNPs, the Hardy–Weinberg equilibrium (HWE) and linkage disequilibrium (LD) were analysed. In this case, the Arlequin v.3.11 and GraphPad Prism (version 5.0) programmes were used, respectively. A significance level was assumed for a value of *p* < 0.05.

## Results

There was a prevalence of males in all groups, especially in patients with cirrhosis and HCC (G1) compared to the group with cirrhosis (G2; *p* = 0.0002) and G3 (*p* = 0.0018). Smoking, alcohol consumption and DM prevailed in patients compared to the controls (*p* < 0.0001; Table 1). Regarding the genetic profile, for *DBP-*rs4588 and *DBP-rs7041*, there was a similarity between groups in all comparisons for genotypic and allelic distribution (*p* > 0.05). For *CYP24A1*-*rs6013897*, the genotype with at least one polymorphic allele (_/T) predominated in G1 (98.3%) versus G2 (88.8%; *p* = 0.0309) (Table 2). HWE was observed for all SNPs, in patients and controls (*p* > 0.05). The LD analysis showed significance for the *rs4588-rs7041* association in patients and controls (*p* = 0.0001, for both). The haplotypes were reconstructed for the set of *DBP* SNPs (*rs4588 and rs7041*) and compared between TotalG and G3, with no significant difference between groups (*p* > 0.05).

Regarding vitamin D, 30% (TGd) of the patients had altered values (<30 ng/mL), and the same was observed in 35% of the individuals without liver disease (G3d); for PTH, altered values (<15 or >65 ng/mL) corresponded to 16.7% and 15%, respectively, with a similarity between groups (*p* > 0.05), for both analyses. Patients with cirrhosis with or without HCC showed a moderate positive correlation (*R*^2^ = 0.3273) between serum vitamin D and PTH values, but this was not observed in the controls (*R*^2^ = 0.0106). Reduced levels of vitamin D in TotalG showed association with genotypes with at least one mutant allele (_/A) for *DBP-rs4588* (77.8%) compared to G3 (14.3%; *p* = 0.0406).

Smoking, alcohol consumption and DM stood out as independent factors for isolated cirrhosis; while for cirrhosis with HCC alcoholism, DM, age, male gender, presence of polymorphic allele of *CYP24A1-rs6013897* (_/T), viral hepatitis and altered serum levels of GGT, AFP and creatinine were the factors (Table 3).

**Figure 1.**
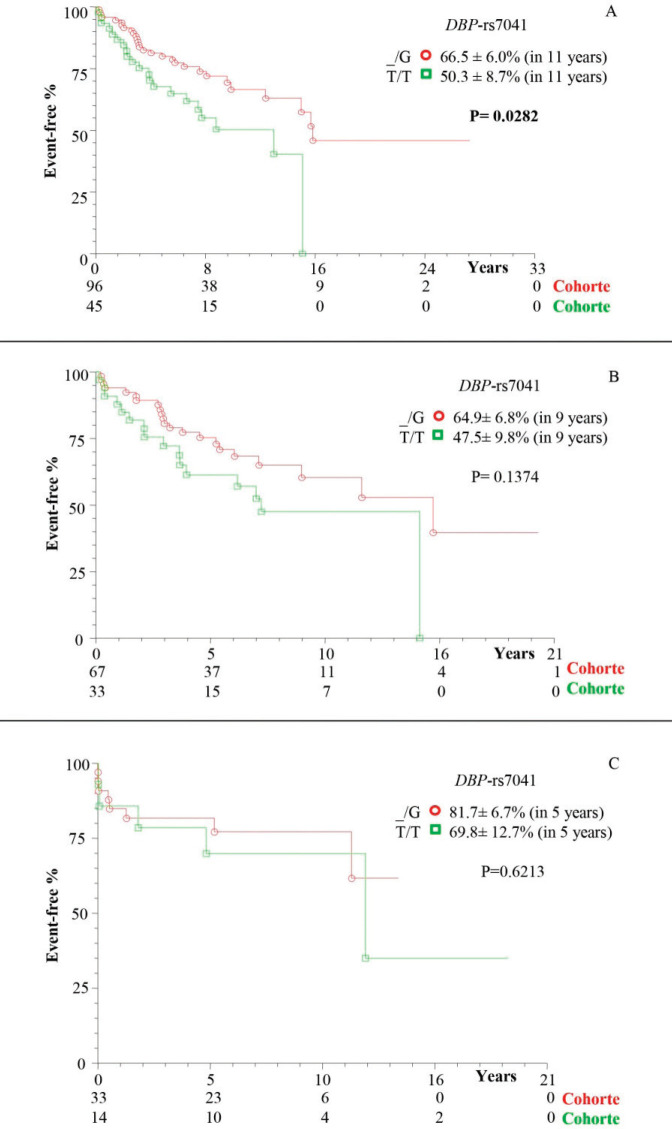
Kaplan–Meier actuarial curve for patients with cirrhosis with or without death event-free HCC, considering the *DBP-rs7041* polymorphism. (a) TotalG of patients (with and without transplant); (b) patients without transplantation; and (c) transplant patients; carriers of genotypes with at least one polymorphic allele (_/G) compared to the wild homozygous genotype (T/T). *DBP *= Vitamin D-binding protein.

**Figure 2.**
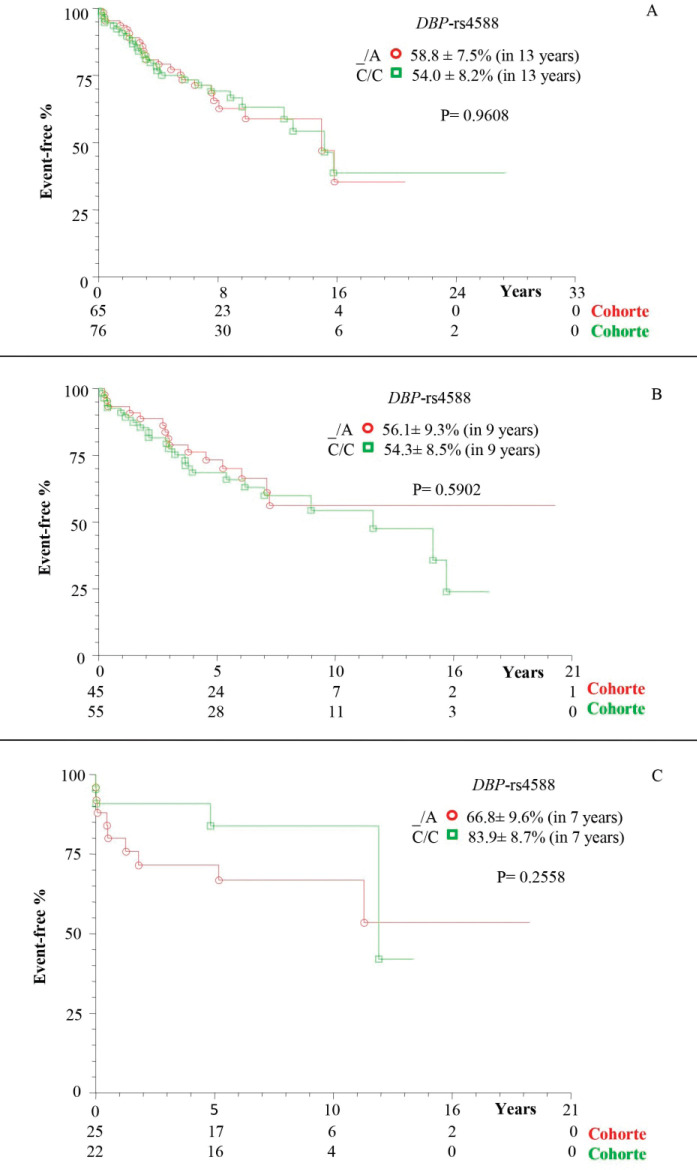
Kaplan–Meier actuarial curve for patients with cirrhosis with or without death event-free HCC, considering the *DBP-rs4588* polymorphism. (a) TotalG of patients (with and without transplant); (b) patients without transplantation; and (c) transplant patients; carriers of genotypes with at least one polymorphic allele (_/A) compared to wild homozygous genotype (C/C). *DBP *= Vitamin D-binding protein.

**Figure 3.**
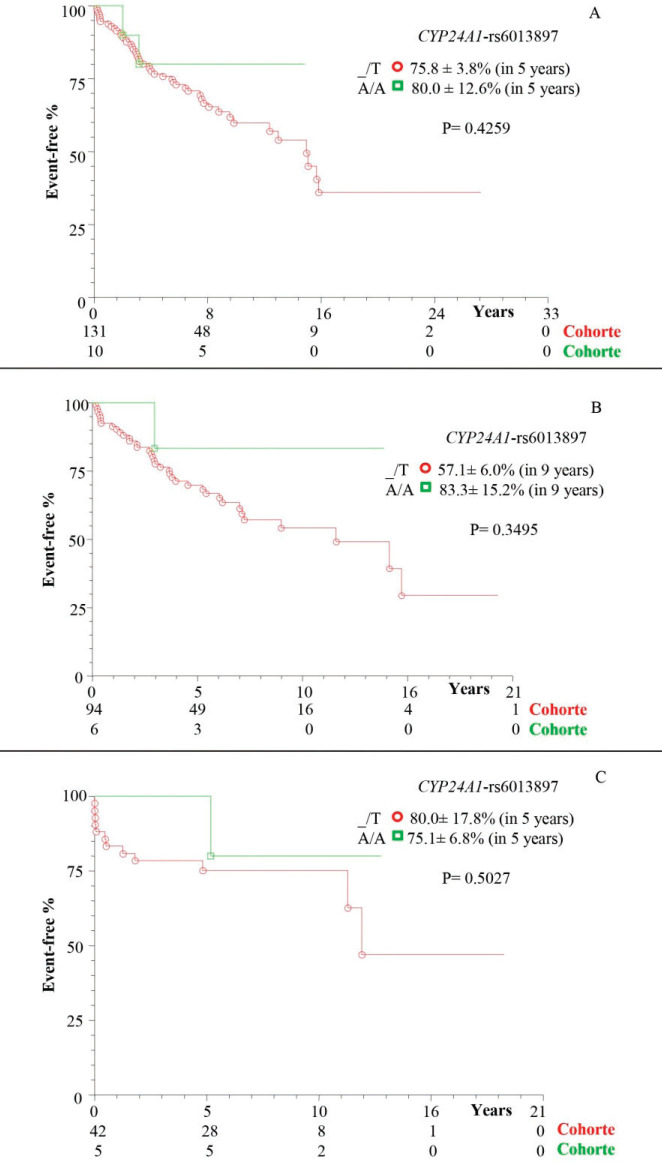
Kaplan–Meier actuarial curve for patients with cirrhosis with or without death event-free HCC, considering the *CYP24A1-rs6013897* polymorphism. (a) TotalG of patients (with and without transplant); (b) patients without transplantation; and (c) transplant patients; carriers of genotypes with at least one polymorphic allele (_/T) compared to the wild homozygous genotype (A/A). *CYP24A1*= Cytochrome P450 family 24 subfamily A member.

There was a higher frequency of survival in patients with at least one polymorphic allele of *DBP*-*rs7041* (_/G = 66.5% ± 6.0%), in 11 years of follow-up, compared to wild homozygote (T/T = 50.3% ± 8.7%; *p* = 0.0282; Figure 1a). However, there was a similarity between the subgroups of patients without transplantation (Figure 1b) and with transplantation (Figure 1c; *p* > 0.05). The same occurred for the polymorphisms *DBP*-*rs4588* (Figure 2a–c) and *CYP24A1*-*rs6013897* (Figure 3a–c) (*p* > 0.05).

## Discussion

Among patients with or without HCC, smoking [27], alcoholism and DM [28] stood out, with a predominance of males [29]. Logistic regression analysis showed these factors as independent risk factors for cirrhosis with or without HCC [30], alcoholism and DM for both, and smoking, particularly in cirrhosis. In addition to cirrhosis with HCC, advanced age and male gender corroborated Mak *et al*’s [29] study. Viral hepatitis [31] and elevated serum creatinine [32] in patients with HCC were also observed in this study.

Admittedly, excessive alcohol intake increases inflammation and oxidative stress [31], causing liver tissue damage with the development of fibrosis, cirrhosis and HCC [33]. Studies show that alcoholism is associated with tobacco addiction, frequent in patients with both cirrhosis and HCC, acting together for the development of the disease, being a potential risk factor for causing DNA damage and thus responsible for hepatocarcinogenesis [31].

In this study, DM also stood out in patients. This metabolic disorder, which is a global public health problem, confers a risk for the development of NAFLD, cirrhosis and HCC [31]. In addition, type 2 DM implies insulin resistance and hyperinsulinemia, causing inflammation, cell proliferation, inhibition of apoptosis and mutation of tumour suppressor genes, which are also responsible for the development of cancer [34].

HWE was confirmed for all SNPs, corroborating the literature [35]. It was also found that LD for *DBP*-*rs4588-DBP-rs7041*, indicating non-random association or co-segregation of these SNPs, was also observed in a series with chronic hepatitis [36], as well as in Brazilian women with polycystic ovary syndrome [37]. The reconstruction of haplotypes for *DBP* showed a similarity between patients and controls, indicating no relationship with risk or protection for the disease, in agreement with a study conducted by Azevedo *et al* [38] in casuistry with chronic hepatitis C.

In this study, patients and controls showed similarity in the genotypic and allelic distribution of *DBP*-*rs4588* and *DBP-rs7041*, as well as in other series [38]. On the other hand, there is a reference to the association of the mutant allele of *DBP-rs7041* (G) with HCC [39] which, however, in this study provided increased survival in patients, with or without transplantation (general group), compared to those with wild genotype (T/T) in 11 years of follow-up, which did not occur when stratified into transplanted and non-transplanted ones. Yet, this polymorphism was associated with decreased survival in patients with lung cancer or kidney disease [40]. In this context, the various factors involved in the pathophysiology must also be considered, as well as the profile of the casuistry.

The effect of genetic variants on the structure and activity of *DBP* stands out, influencing its affinity to vitamin D, as well as the concentration of 25(OH)D, as reported for the *DBP* polymorphisms *rs7041* and *rs458836*. Incidentally, this study showed a higher frequency of reduced vitamin D levels in patients with cirrhosis with or without HCC carrying at least one mutant allele (_/A) for* DBP-rs4588*, compared to the control group, in agreement with Santos *et al* [37]. There is also a reference to the influence of the polymorphisms of *DBP-rs7041* and *DBP*-rs4588 affecting the binding affinity of *DBP* with vitamin D, which could consequently influence its anti-tumorigenesis activity [41]. However, haplotype variations in different ethnicities may contribute to divergences between studies [39, 42].

In this study, *CYP24A1-rs6013897* (_/T) polymorphism was also associated with cirrhosis and HCC, as an independent factor for the disease, while the wild homozygous genotype (A/A) prevailed in controls. There is reference to the association of the *CYP24A1-rs6013897* polymorphism with colorectal cancer [43], in addition to inflammatory reactions, however, there are few studies on cirrhosis with or without HCC [21], thus highlighting the contribution of the present study.

In the logistic regression analysis, GGT was identified as an independent factor in the worsening of HCC. The GGT enzyme is a serum marker found in hepatic and biliary cells. Elevated serum levels are associated with an increased risk of developing liver, metabolic, cardiovascular and diabetes diseases [44]. Additionally, increased AFP values prevailed in patients with cirrhosis and HCC, compared to the group with cirrhosis, corroborating the literature [45], also presenting itself as an independent factor for the disease. Widely used in the diagnosis of liver cancer, AFP is considered an important serological marker. However, it is advisable to use this marker only as a preventive and non-diagnostic method for HCC [46], considering that high levels of AFP can also be found in chronic liver disease [45].

Regarding vitamin D, there was similarity between the groups; however, another casuistry showed an association of reduced levels of vitamin D with various types of cancer [47]. Furthermore, a cohort study showed vitamin D deficiency associated with advanced stages of the disease and mortality [48]. In the present study, approximately 30% of the patients and controls showed serum vitamin D levels <30 ng/mL. Nevertheless, it is reported that about two-thirds of patients with HCC present vitamin D deficiency, showing that serum levels are inversely correlated with the progression of liver diseases [49].

A Brazilian epidemiological study showed reduced levels of vitamin D in the southeast region of the country [50]. However, the Brazilian population of mixed ethnicity and the fluctuation of 25(OH)D_3_ levels during the seasons of the year must be considered [51]. In addition to that, the restricted number of patients for vitamin D analysis, although it did not prevent the statistical analysis of the data, was a limiting factor in this study.

The correlation analysis between vitamin D and PTH levels was also carried out, considering that even in situations of vitamin D deficiency, the active form [1,25(OH)2D] can present satisfactory levels under the mediation of PTH [52]. In this process, PTH acts on the bone to increase the influx of calcium and phosphate, with the purpose of increasing the synthesis of vitamin D, which, in turn, suppresses the production and secretion of PTH, ending the negative feedback cycle [53].

In this study, patients and controls showed similarity in PTH levels, corroborating a study in breast cancer [54]. On the other hand, vitamin D and PTH showed a moderate positive correlation only in patients, while in another casuistry [55], a negative correlation was observed, suggesting high PTH values in vitamin D deficiency and vice versa. In this context, it is noteworthy that genetic alterations in *DBP*, which actively participate in the vitamin D metabolic pathway, could influence its serum level, with compensatory PTH secretion in case of vitamin D deficiency [52], which may have occurred in the presence of *DBP-rs4588* which in this series was associated with reduced levels of vitamin D.

## Conclusion

In conclusion, the *CYP24A1-rs6013897* polymorphism is associated with cirrhosis and HCC, standing out as a predictor of the disease, while the *DBP-rs4588* polymorphism can influence in the reduction of vitamin D level in patients and, on the other hand, the variant *DBP*-*rs7041* confers increased survival, suggesting a protective character. Age, alcoholism, DM, viral hepatitis and high levels of GGT, AFP and creatinine are also confirmed as predictors of both diseases, while smoking, alcoholism and DM are predictors of isolated cirrhosis.

## Conflicts of interest

There are no conflicts of interest to declare.

## Figures and Tables

**Table 1.** Demographic profile, lifestyle and comorbidities in the TotalG of patients, patients with cirrhosis and HCC (G1), with isolated cirrhosis (G2) and individuals without the disease (G3).

Characteristic	TotalG (*N* = 158)		G1 (*N* = 60)		G2 (*N* = 98)		G3 (*N* = 225)		*p-value			
									TotalG × G3	G1 × G2	G1 × G3	G﻿2 × G3
Sex	*N*	%	*N*	%	*N*	%	*N*	%				
Male	124	78.5	50	83.3	74	75.5	127	56.4	**<0.0001**	**0.0002**	**0.0018**	0.3361
Female	34	21.5	10	16.7	24	24.5	98	43.6				
												
Lifestyle	*N*	%	*N*	%	*N*	%	*N*	%				
Tobacco	74	46.8	27	45.0	47	48.0	45	20.0	**<0.0001**	0.8434	**<0.0001**	**<0.0001**
Alcohol	98	62.0	39	65.0	59	60.2	26	11.6	**<0.0001**	0.6643	**<0.0001**	**<0.0001**
Comorbidity	*N*	%	*N*	%	*N*	%	*N*	%				
DM	41	26.0	16	26.7	25	25.5	8	3.6	**<0.0001**	0.8721	**<0.0001**	**<0.0001**
SAH	53	33.5	18	30.0	35	35.7	70	31.1	0.6959	0.5723	0.9934	0.4947

*p* = Significance level

* Chi-square or Fisher test; significance level = *p* < 0.05

*N* = Number of individuals; DM = Diabetes mellitus; SAH = Systemic arterial hypertension

**Table 2.** Allelic and genotypic frequencies for the DBP (rs4588 and rs7041) and CYP24A1 (rs6013897) polymorphisms in the TotalG of patients, patients with cirrhosis and HCC (G1), patients with isolated cirrhosis (G2) and individuals without the disease (G3).

Genetic profile	TotalG *N* = 158		G1 *N* = 60		G2 *N* = 98		G3 *N* = 225		*p-value			
									TotalG × G3	G1 × G2	G1 × G3	G2 × G3
DBP (rs4588) Genotype	*N*	%	*N*	%	*N*	%	*N*	%				
C/C	76	48.1	27	45.0	49	50.0	119	52.9	0.4129	0.6553	0.3468	0.7214
C/A	68	43.0	25	41.7	43	43.9	86	38.2	0.4007	0.9149	0.7360	0.4063
A/A	14	8.9	8	13.3	6	6.1	20	8.9	0.9924	0.2078	0.4333	0.5368
_/A	82	51.9	33	55.0	49	50.0	106	47.1	0.4129	0.6553	0.3468	0.7214

Allele	*N*	AF	*N*	AF	*N*	AF	*N*	AF				
C	220	0.70	79	0.65	141	0.72	324	0.72	0.5262	0.3081	0.2278	0.9873
A	96	0.30	41	0.35	55	0.28	126	0.28				

DBP (rs7041) Genotype	*N*	%	*N*	%	*N*	%	*N*	%				
T/T	52	32.9	19	31.7	33	33.7	57	25.3	0.1328	0.9314	0.4114	0.1609
T/G	67	42.4	29	48.3	38	38.8	105	46.7	0.4708	0.3106	0.9328	0.2337
G/G	39	24.7	12	20.0	27	27.6	63	28.0	0.5449	0.3798	0.2777	0.9341
_/G	106	67.1	41	68.3	65	66.3	168	74.7	0.1328	0.9314	0.4114	0.1609

Allele	*N*	AF	*N*	AF	*N*	AF	*N*	AF				
T	171	0.54	67	0.56	104	0.53	219	0.49	0.1582	0.7161	0.1962	0.3465
G	145	0.46	53	0.44	92	0.47	231	0.51				

CYP24A1 (rs6013897) Genotype	*N*	%	*N*	%	*N*	%	*N*	%				
A/A	12	7.6	1	1.7	11	11.2	17	7.6	0.9886	**0.0309**	0.1347	0.3885
A/T	65	41.1	28	46.7	37	37.7	79	35.1	0.2749	0.3481	0.1356	0.7420
T/T	81	51.3	31	51.7	50	51.0	129	57.3	0.2845	0.9371	0.5225	0.3536
_/T	146	92.4	59	98.3	87	88.8	208	92.6	0.9886	**0.0309**	0.1347	0.3885

Allele	*N*	AF		AF	*N*	AF	*N*	AF				
A	89	0.28	30	0.25	59	0.30	113	0.25	0.3893	0.3955	0.9801	0.2215
T	227	0.72	90	0.75	137	0.70	337	0.75				

*DBP* = Vitamin D-binding protein; *CYP24A1* = Cytochrome P450 family 24 subfamily A member 1; A/A = Wild homozygous genotype; A/T = Heterozygous genotype; T/T = mutant homozygous genotype; *N* = Number of individuals; AF = Absolute frequency

*p* = Significance level; significance level = *p* < 0.05

* Chi-Square or Fisher’s test

**Table 3.** Risk profile by logistic regression analysis in a TotalG of patients (TotalG = cirrhosis with or without HCC), with cirrhosis and HCC (G1), with isolated cirrhosis (G2) and subgroups with vitamin dosage D (TGd, G1d and G2d), compared to individuals without liver disease.

	TotalG			G1			G2			G1 × G2		
Covariates	ratio	Confidence interval	*p-value	Odds ratio	Confidence interval	*p-value	Odds ratio	Confidence interval	*p-value	Odds ratio	Confidence interval	*p-value
Tobacco	2.05	1.16–3.63	0.0129	-	-	**-**	2.50	1.34–4.65	0.004	-	-	-
Alcohol	12.68	7.13–22.56	<0.0001	24.36	10.25–57.91	<0.0001	10.51	5.65–19.57	<0.0001	-	-	-
DM	11.11	4.58–26.97	<0.0001	15.27	4.53–51.47	<0.0001	10.04	3.85–26.17	<0.0001	-	-	-
SAH	-	-	-	-	-	-	-	-	-	-	-	-
Age	1.03	1.01–1.05	0.0031	1.08	1.04–1.12	<0.0001	-	-	-	1.08	1.03–1.13	0.0005
Male	-	-	**-**	-	-	**-**	-	-	-	3.36	1.17–9.59	0.0237
*DBP*-rs4588 _/A	-	-	-	-	-	-	-	-	-	-	-	-
*DBP*-rs7041_/G	-	-	**-**	-	-	-	-	-	**-**	-	-	-
*CYP24A1*-rs6013897 _/T	-	-	-	-	-	-	-	-	-	14.18	1.50–133.71	0.0205
Viral Hepatitis	-	-	-	-	-	-	-	-	-	3.09	1.22–7.81	0.0169
GGT	-	-	-	-	-	-	-	-	-	3.85	1.40–10.58	0.009
AFP	-	-	-	-	-	-	-	-	-	5.46	2.32–12.88	0.0001
Creatinine	-	-	-	-	-	-	-	-	-	3.58	1.41–9.07	0.0071
		**TGd**			**G1d**			**G2d**			**G1d × G2d**	
**Covariates**	**Odds ratio**	**Confidence interval**	*p-value	**Odds ratio**	**Confidence interval**	*p-value	**Odds ratio**	**Confidence interval**	*p-value	**Odds ratio**	**Confidence interval**	*p-value
Tobacco	-	-	**-**	14.50	1.10–189.46	0.0414	-	-	**-**	-	-	-
Alcohol	21.68	2.56–183.21	0.0047	12.10	1.09–1.10	0.0421	-	-	**-**	-	-	-

* Significance level = *p* < 0.05

DM = Diabetes mellitus; SAH = Systemic arterial hypertension; *DBp* = Vitamin D-binding protein; *CYP24A1* = Cytochrome P450 family 24subfamily A member 1; GGT = gamma-glutamyl transferase, AFP = alpha-fetoprotein, d = subgroup with vitamin D dosage
